# Supplementary material for: The Long Noncoding RNA LOC441461 (STX17-AS1) Modulates Colorectal Cancer Cell Growth and Motility
Source: Cancers (Basel). 2020 Oct 28;12(11):3171. doi: 10.3390/cancers12113171 (PMC7692211; doi:10.3390/cancers12113171)
Supplement: Supplementary file 1 [file cancers-12-03171-s001.pdf]

cancers

Article

The Long Noncoding RNA LOC441461 (STX17-AS1) Modulates Colorectal Cancer Cell Growth and Motility

Jui-Ho Wang, Tzung-Ju Lu, Mei-Lang Kung, Yi-Fang Yang, Chung-Yu Yeh, Ya-Ting Tu , Wei-Shone Chen and Kuo-Wang Tsai

Supplementary Materials

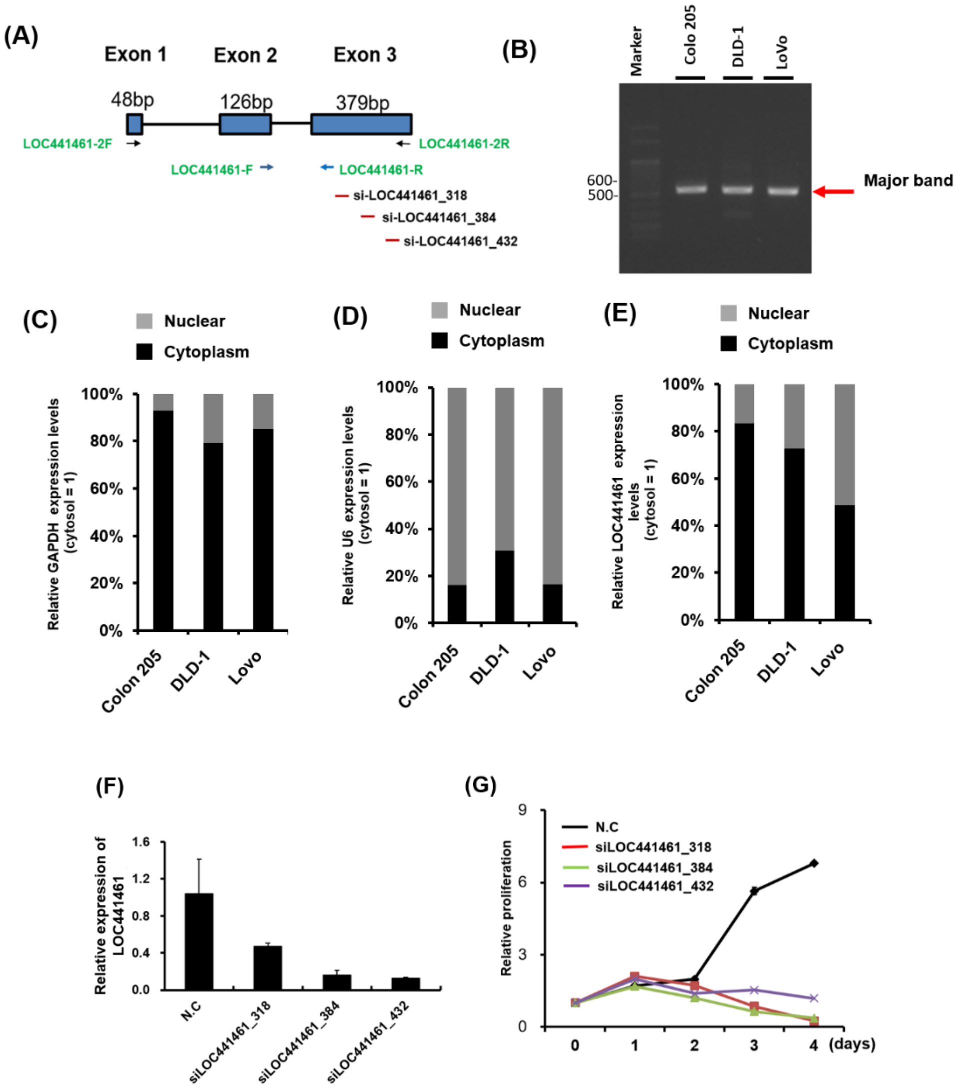

Figure S1. LOC441461 knockdown suppressed colon cancer growth in SW620 cells. (**A**) Schema of the locations of four PCR primers used for LOC441461 amplification and three siRNA sequences designed for LOC441461 knockdown in this study. The gene structure of LOC441461 was obtained from the human genome database of the University of California, Santa Cruz. (**B**) The putative transcript of LOC441461 was identified using PCR with the LOC441461-2F/-2R primer in this study. (**C**,**D**,**E**) RNA expression levels of GAPDH, U6, and LOC441461 candidates, respectively, in the nucleus and cytoplasm were measured using RT-PCR. GAPDH acted as a marker for the cytoplasm, and U6 acted as a nuclear marker. (**F**) After transfection with individual siRNA (si-LOC441461_318,

si-LOC441461_384, and si-LOC441461_432), LOC441461 expression levels were examined using RT-PCR. (**G**) Cell proliferation was assessed in the SW620 cells with LOC441461 knockdown by using three individual siRNAs.

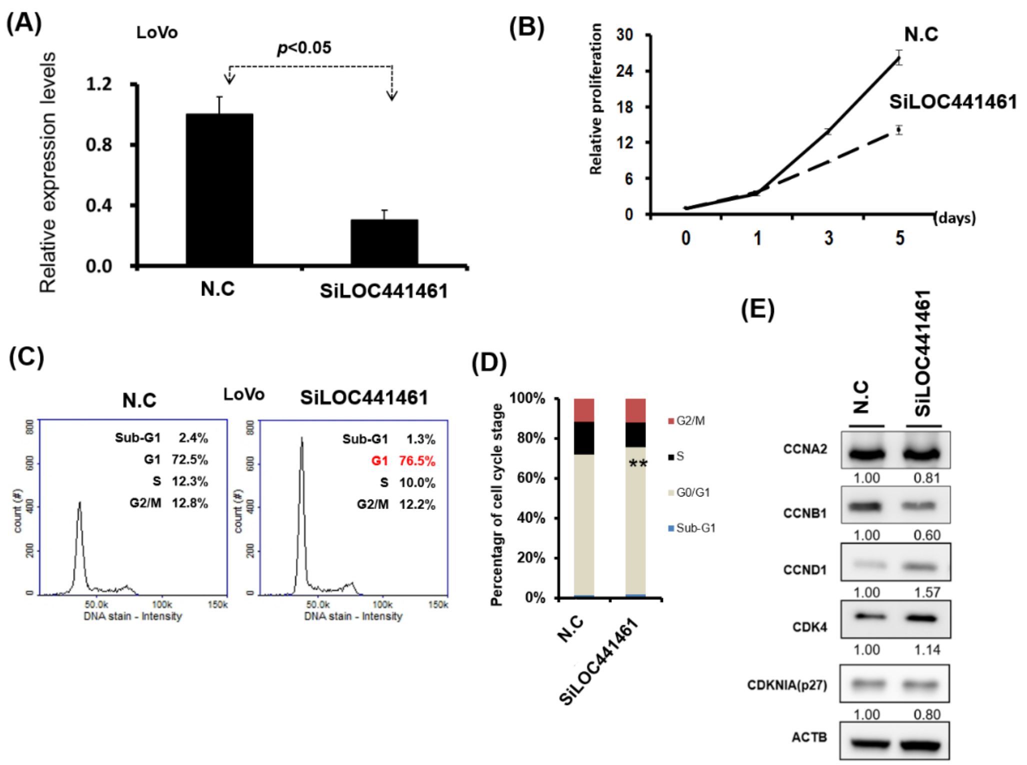

Figure S2. LOC441461 knockdown suppressed LoVo cell growth by impairing cell cycle progression. (**A**) LOC441461 expression levels were examined using RT-PCR in LoVo cells with si-LOC441461 transfection. (**B**) Cell proliferation compared with the scrambled control was measured using the CellTiter-Glo One Solution assay at various time points (0, 1, 3, and 5 days). (**C**) The distribution of cells in three phases of the cell cycle was examined using the image flow cytometry assay. (**D**) Graph of each quantified phase. (**E**) Cell-cycle-related protein levels were examined using the Western blotting assay in LoVo cells with and without LOC441461 knockdown. All experiments were performed in triplicate, and these data were analyzed using Student’s t test. The difference was considered significant when $p < 0.05$.

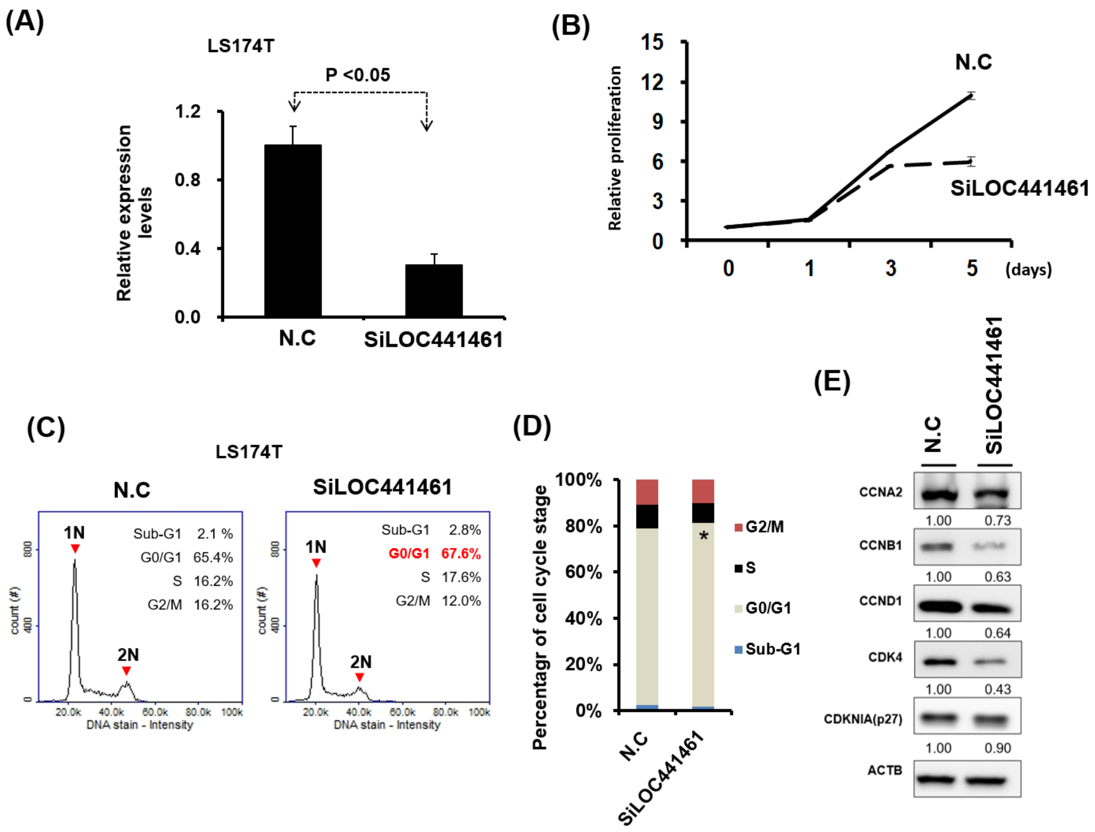

Figure S3. LOC441461 knockdown suppressed LS174T cell growth by impairing cell cycle progression. (**A**) LOC441461 expression levels were examined using RT-PCR in LS174T cells with si-LOC441461 transfection. (**B**) Cell proliferation compared with the scrambled control was measured using the CellTiter-Glo One Solution assay at various time points (0, 1, 3, and 5 days). (**C**) The distribution of cells in three phases of the cell cycle was examined using the image flow cytometry assay. (**D**) Graph of each quantified phase. (**E**) Cell-cycle-related protein levels were examined using the Western blotting assay in LS174T cells with and without LOC441461 knockdown. All experiments were performed in triplicate, and these data were analyzed using Student's t test. The difference was considered significant when $p < 0.05$.

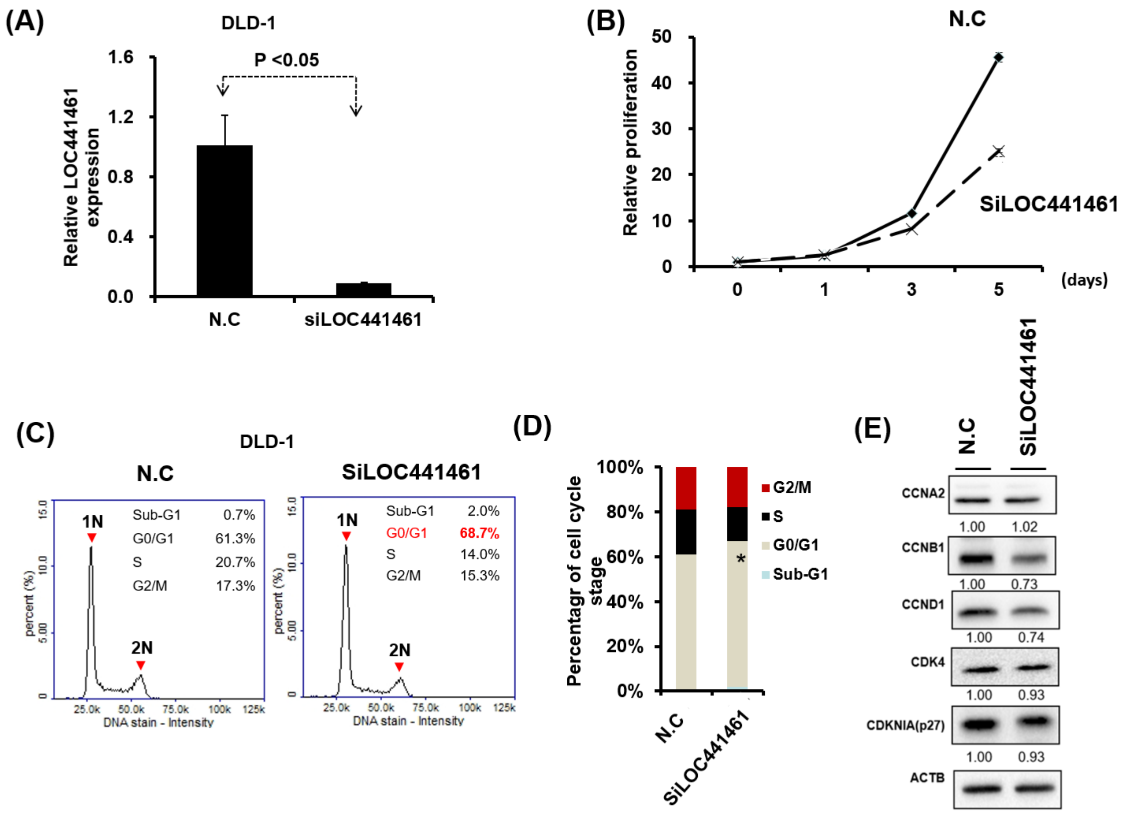

Figure S4. LOC441461 knockdown suppressed DLD-1 cell growth by impairing cell cycle progression. (**A**) LOC441461 expression levels were examined using RT-PCR in DLD-1 cells with si-LOC441461 transfection. (**B**) Cell proliferation compared with the scrambled control was measured using the CellTiter-Glo One Solution assay at various time points (0, 1, 3, and 5 days). (**C**) The distribution of cells in three phases of the cell cycle was examined using the image flow cytometry assay. (**D**) Graph of each quantified phase. (**E**) Cell-cycle-related protein levels were examined using the Western blotting assay in DLD-1 cells with and without LOC441461 knockdown. All experiments were performed in triplicate, and these data were analyzed using Student's t test. The difference was considered significant when $p < 0.05$.

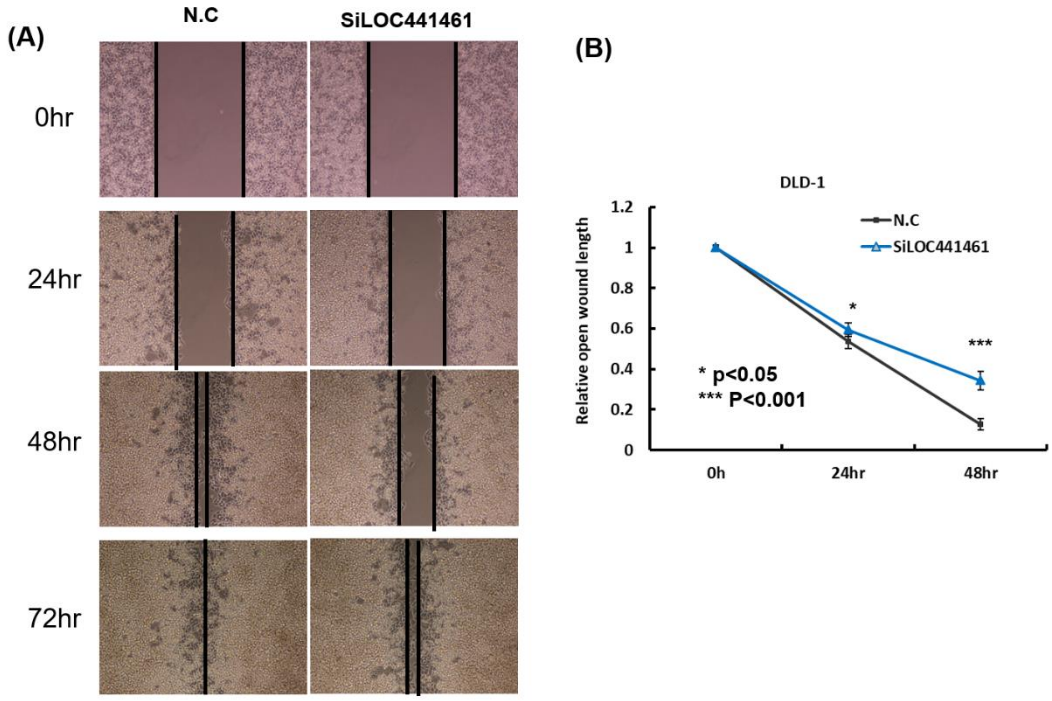

Figure S5. LOC441461 knockdown suppressed DLD-1 cell wound healing ability. (**A**) The wound healing assay was performed in DLD-1 cells transfected with siLOC441461 and the scrambled control (N.C). (**B**) The relative migration ability was quantified by calculating the open wound length.

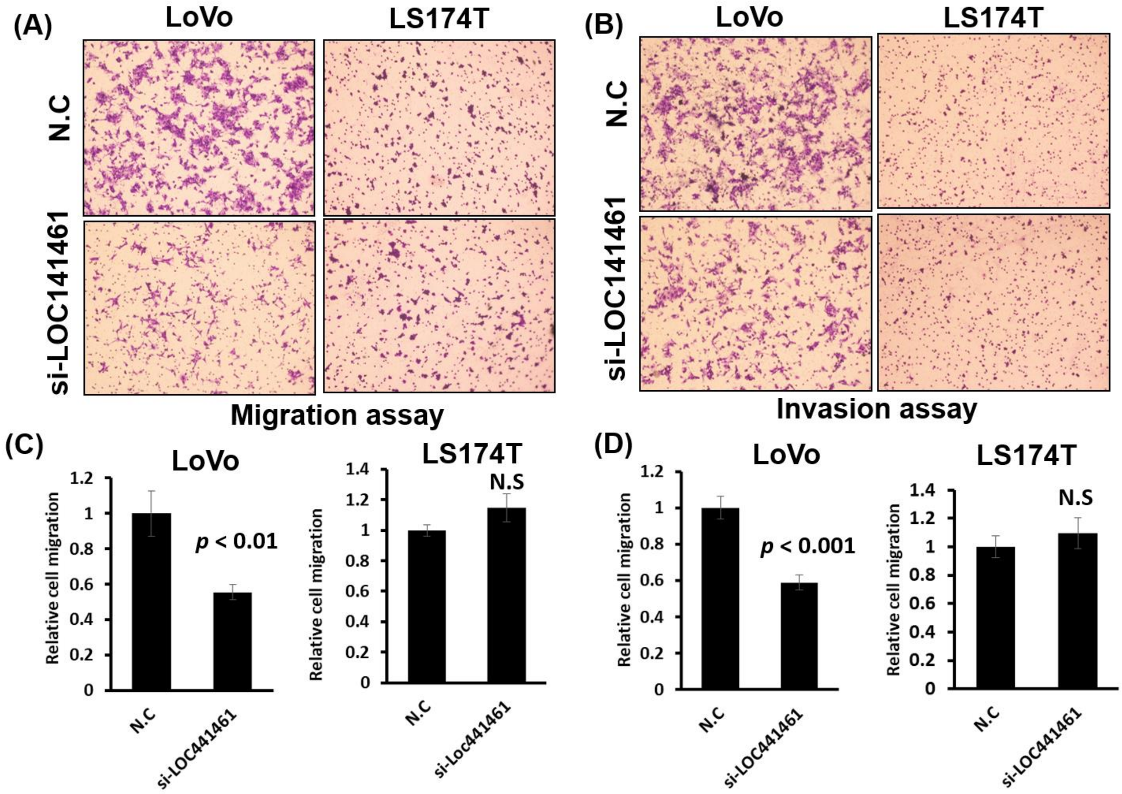

Figure S6. Effects of LOC441461 knockdown on the invasion and migration abilities of LoVo and LS174T cells. (**A**) and (**B**) After the knockdown of LOC441461 in LoVo and LS174T cells, their migration and invasion abilities were assessed using the transwell assay. (**C**) and (**D**) The migration and invasion abilities were further quantified using the Ascent software. The difference was considered significant when $p < 0.05$. All experiments were performed in triplicate, and these data

were analyzed using Student's t test. The difference was considered significant when $p < 0.05$. N.S. = not significant.

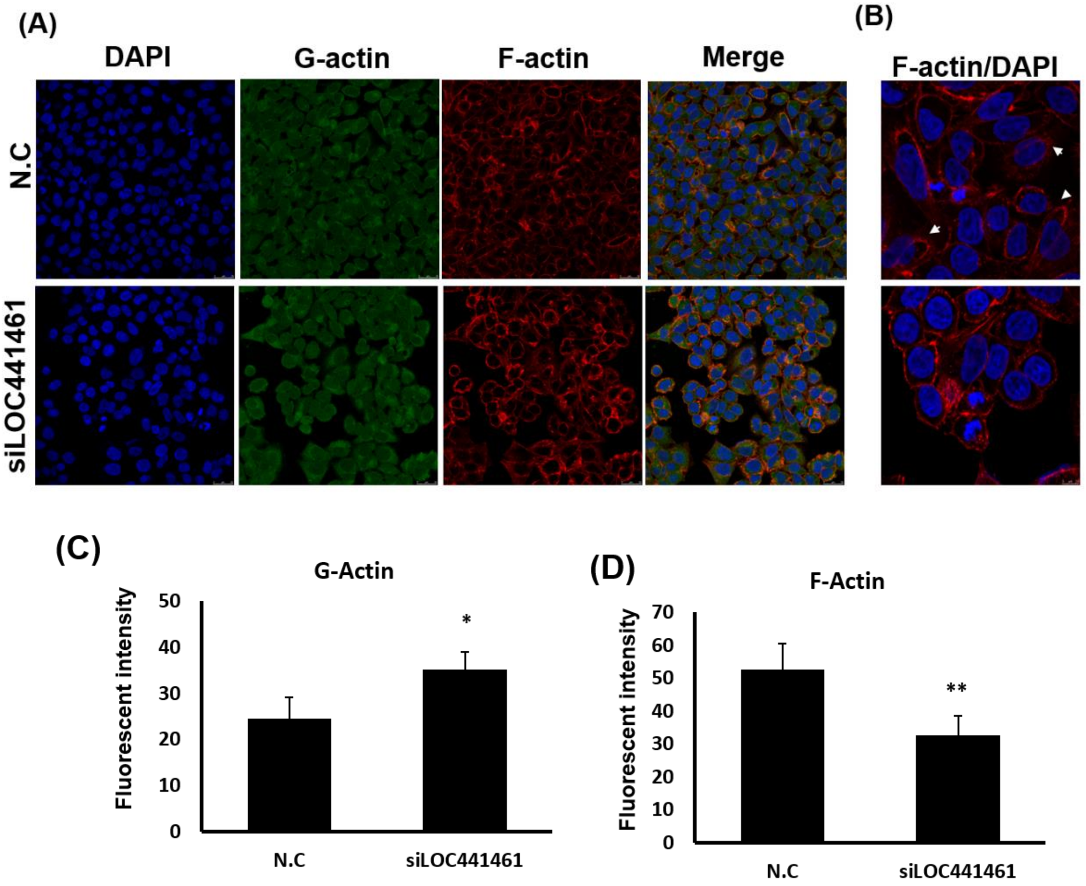

Figure S7. LOC441461 knockdown suppressed colon cancer cell motility through G-actin and F-actin polymeration. (**A**) DLD-1 cells treated with or without siLOC441461 siRNA were stained with rhodamine phalloidin, Alexa Fluor 488 DNase I conjugate, and 4′,6-diamidino-2-phenylindole for the detection of F-actin (red), G-actin (green), and nuclear (blue). (**B**) F-actin labeling with rhodamine phalloidin revealed that DLD-1 cells exhibited numerous filopodia, and the LOC441461-knockdown cells exhibited fewer filopodium fibers than the DLD-1 cells did. (**C**) and (**D**) The fluorescent intensity of G-actin and F-actin was calculated using a confocal microscope ($n = 3$).

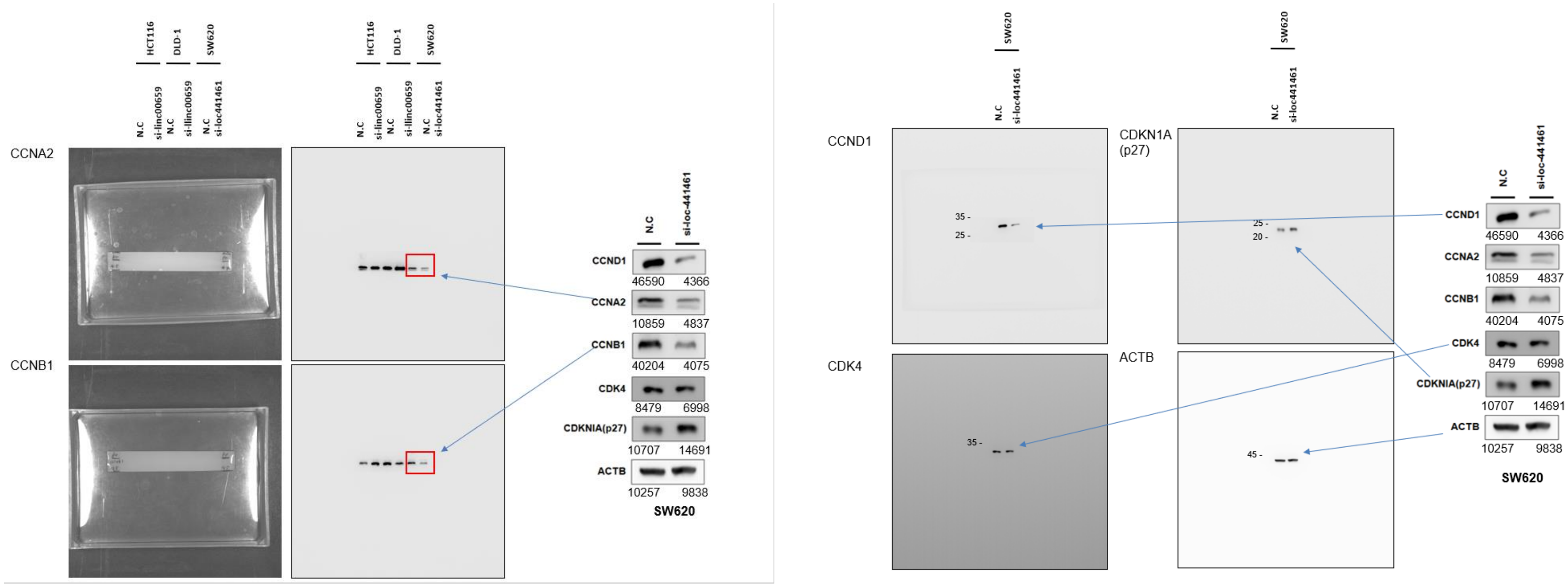
HCT116
DLD-1
SW620
N.C
si-linc00659
N.C
si-linc00659
N.C
si-loc441461
CCNA2
CCNB1
N.C
si-loc-441461
CCND1
46590
4366
CCNA2
10859
4837
CCNB1
40204
4075
CDK4
8479
6998
CDKNIA(p27)
10707
14691
ACTB
10257
9838
SW620
CCND1
CDKN1A (p27)
CDK4
ACTB
35 -
25 -
20 -
45 -

(A)

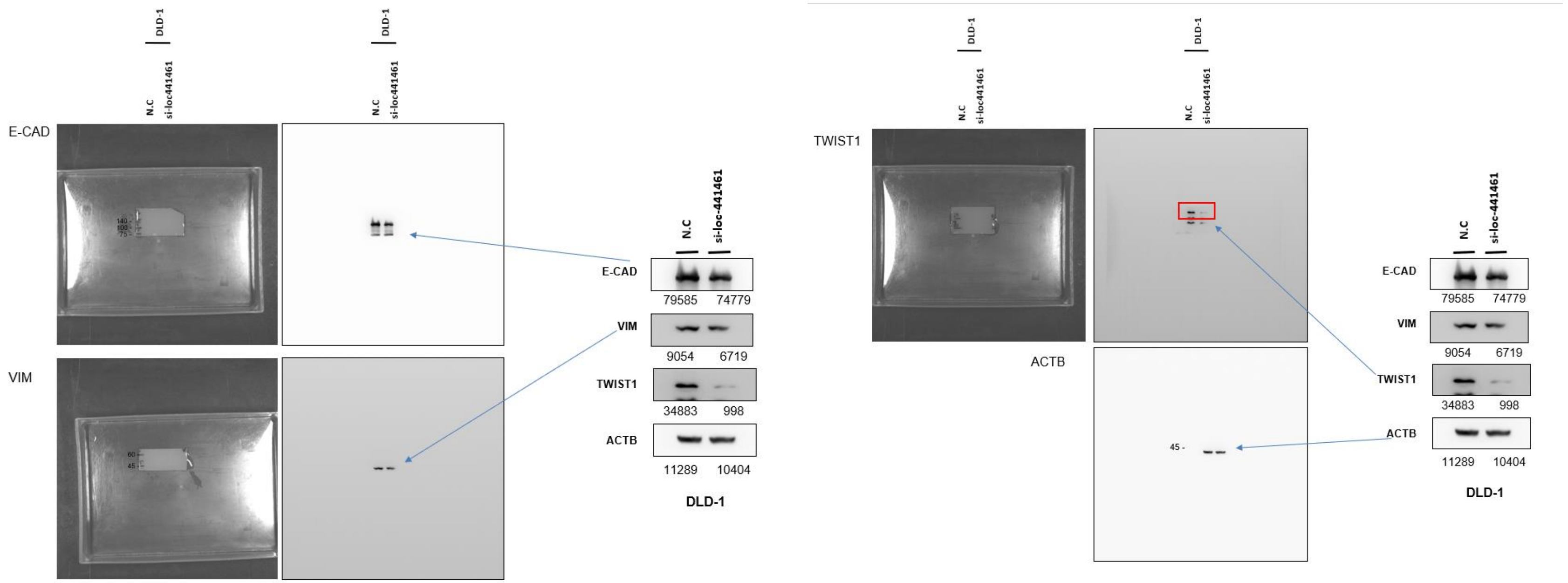
DLD-1
N.C
si-loc441461
E-CAD
VIM
TWIST1
ACTB
45 -
N.C
si-loc-441461
E-CAD
79585 74779
VIM
9054 6719
TWIST1
34883 998
ACTB
11289 10404
DLD-1

(B)

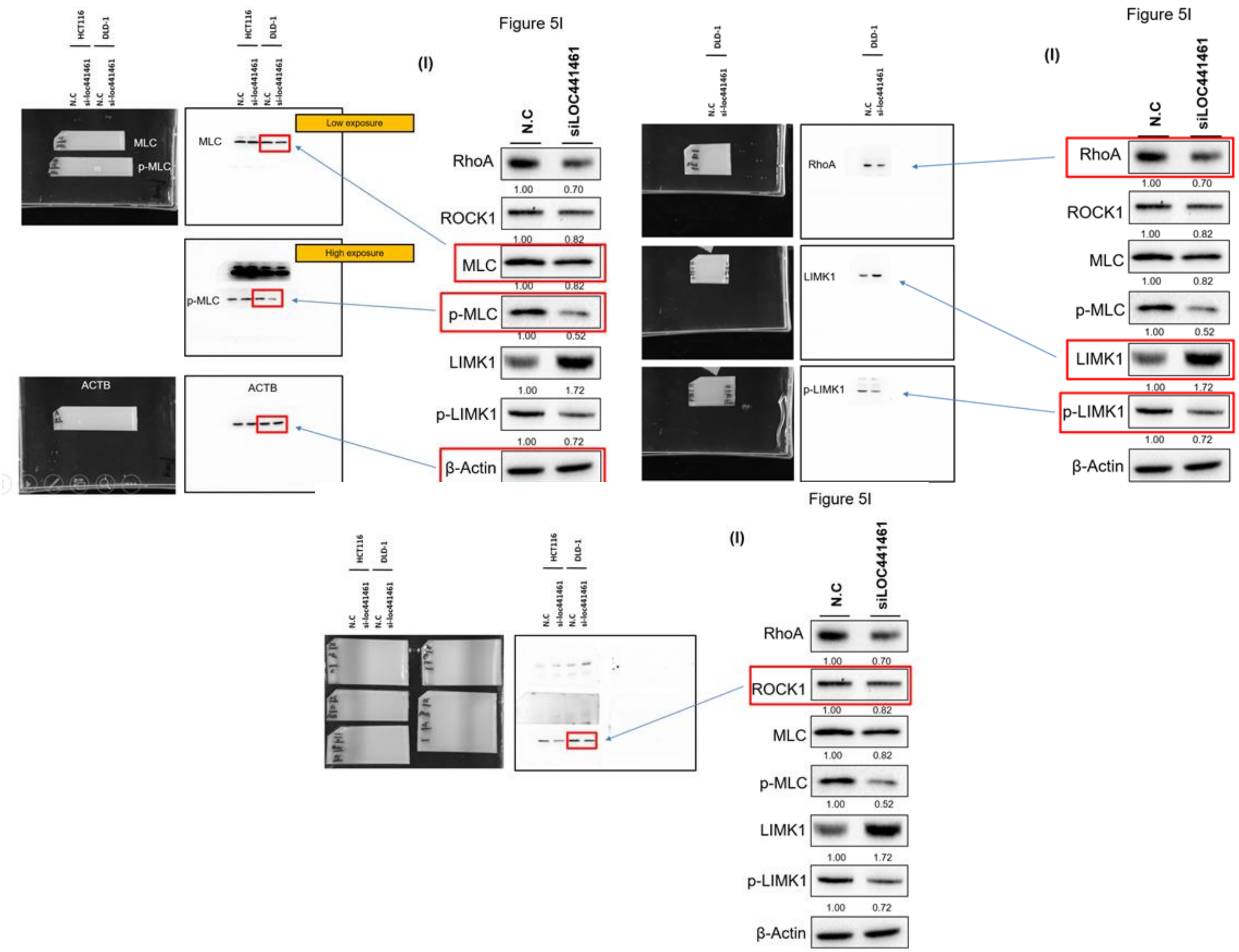

(C)

Figure S8. Whole Western Blots. (**A**) Figure 4D (**B**) Figure 5G (**C**) Figure 5I.

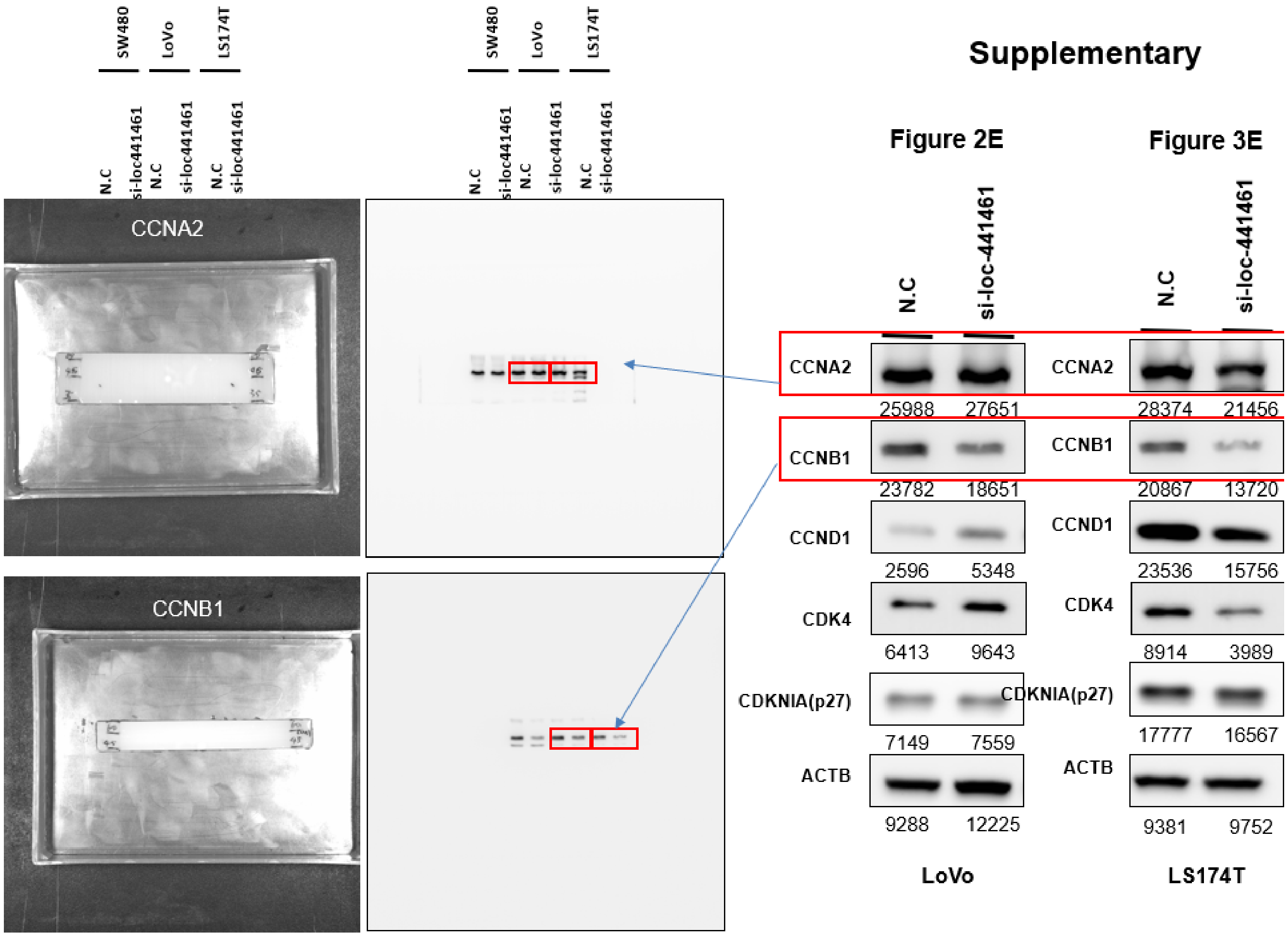
SW480
LoVo
LS174T
N.C
si-loc441461
N.C
si-loc441461
N.C
si-loc441461
CCNA2
CCNB1
Supplementary
Figure 2E
Figure 3E
N.C
si-loc-441461
N.C
si-loc-441461
CCNA2
25988 27651
CCNA2
28374 21456
CCNB1
23782 18651
CCNB1
20867 13720
CCND1
2596 5348
CCND1
23536 15756
CDK4
6413 9643
CDK4
8914 3989
CDKNIA(p27)
7149 7559
CDKNIA(p27)
17777 16567
ACTB
9288 12225
ACTB
9381 9752
LoVo
LS174T

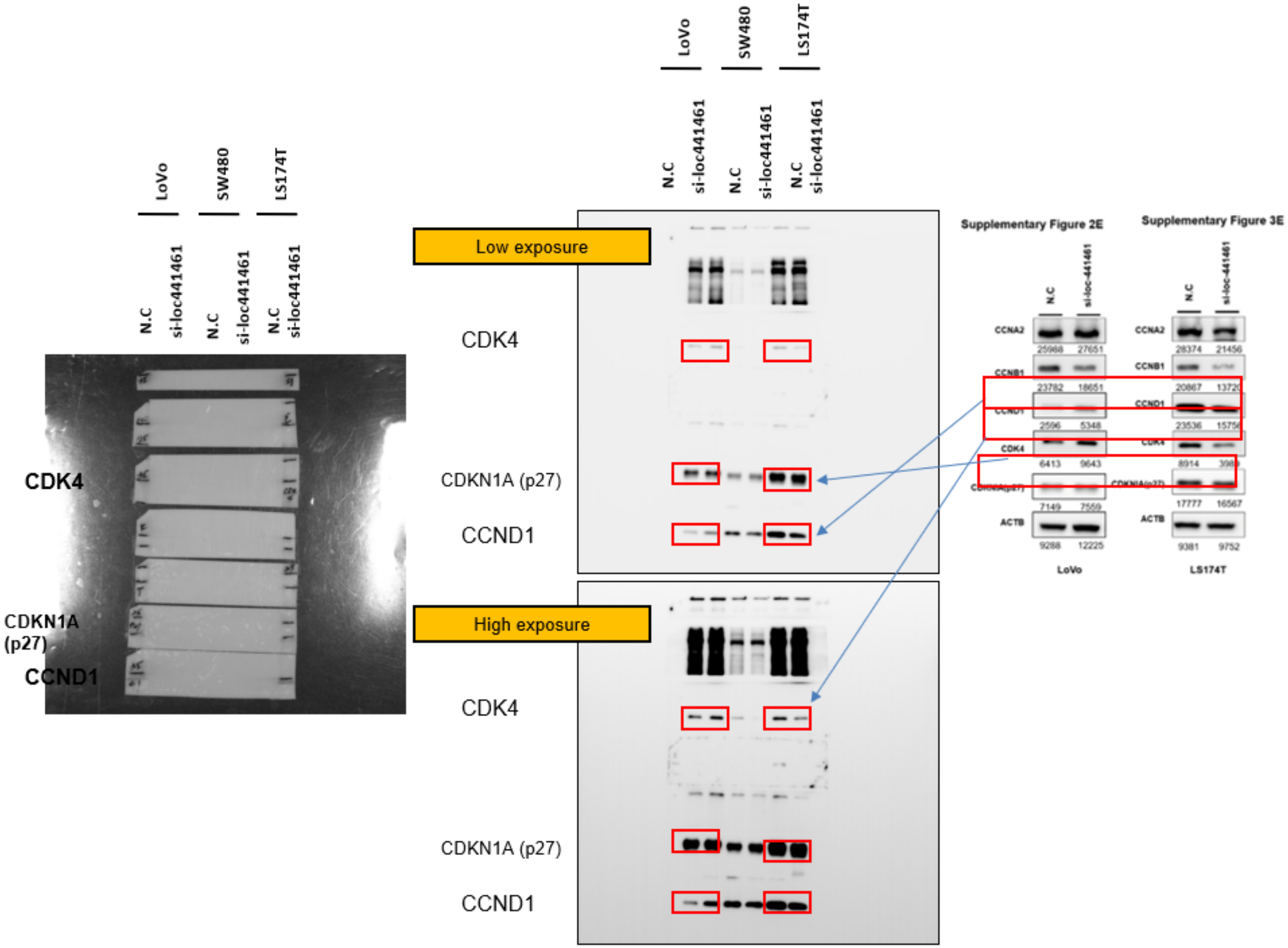

LoVo
SW480
LS174T
N.C
si-loc441461
N.C
si-loc441461
N.C
si-loc441461
CDK4
CDKN1A
(p27)
CCND1
Low exposure
CDK4
CDKN1A (p27)
CCND1
High exposure
CDK4
CDKN1A (p27)
CCND1
Supplementary Figure 2E
Supplementary Figure 3E
LoVo
LS174T

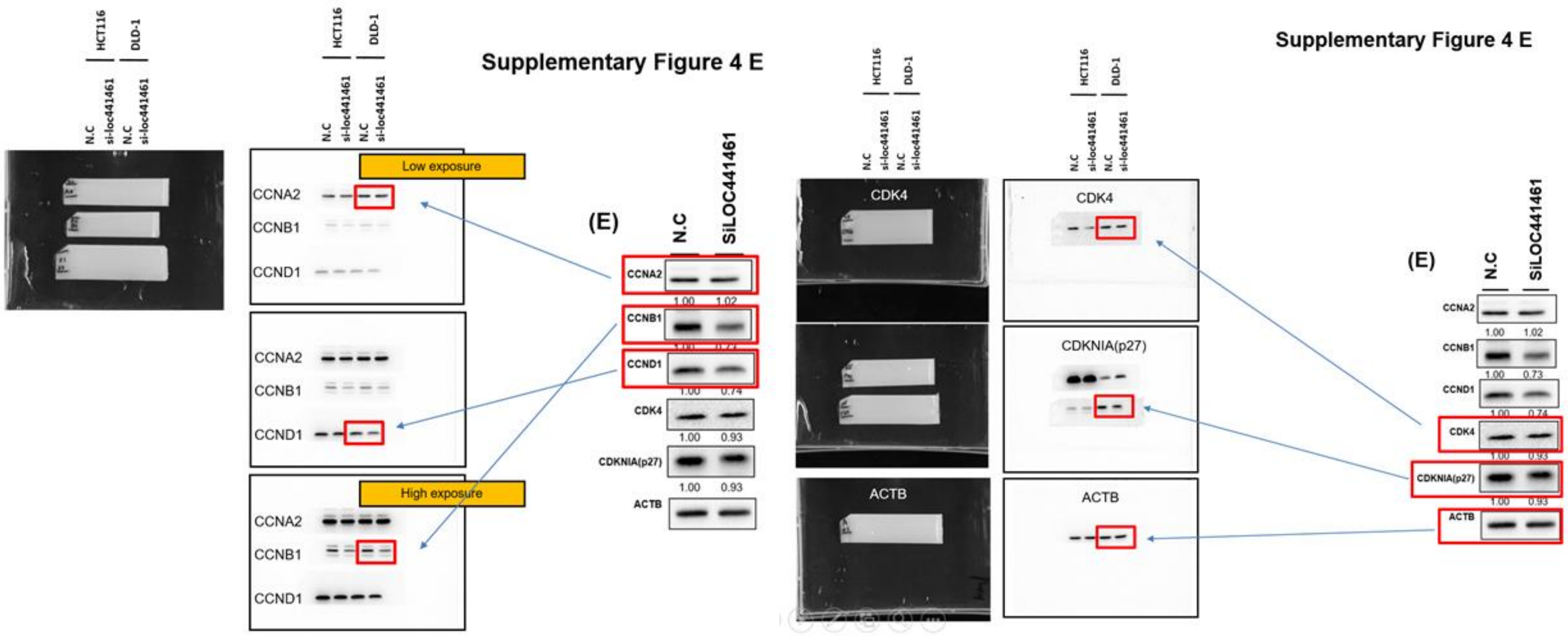

Figure S9. Whole Western Blots for Supplementary Figures.

Table S1. Results of the pathway enrichment analysis of the differentially expressed genes.

Gene Ontology	Signaling Pathway	*p* Value
Biological process	protein targeting to mitochondrion	4.79×10^{-5}
	protein transport	0.00931
	regulation of cell shape	0.009792
	intracellular protein transport	0.013446
	cellular response to nerve growth factor stimulus	0.014592
	small GTPase mediated signal transduction	0.015829
	transferrin transport	0.018386
	COPI coating of Golgi vesicle	0.023611
	positive regulation of cholesterol storage	0.040956
	cellular response to laminar fluid shear stress	0.04667
	macropinocytosis	0.04667
	regulation of Golgi organization	0.04667
	G2/M transition of mitotic cell cycle	0.048495
Cellular component	Golgi membrane	1.38×10^{-4}
	Golgi apparatus	0.00125
	intracellular	0.001381
	endoplasmic reticulum-Golgi intermediate compartment membrane	0.005845
	membrane	0.005856
	endosome	0.009396
	intracellular membrane-bounded organelle	0.040597
Molecular function	protein binding	0.004797
	protein kinase binding	0.020343
	GTPase activator activity	0.021513
	GTP binding	0.02229
	SH3 domain binding	0.030391

Text S1: Supplementary Materials and Methods

Immunofluorescence of Actin Staining

DLD-1 cells with LOC441461 knockdown were seeded on coverslips. The cells were fixed for 15 min at room temperature in 2.0% paraformaldehyde in PBS, washed thrice with PBS, and then permeabilized for 10 minutes at room temperature in PBS containing 0.1% Triton X-100. The cells were labeled with rhodamine phalloidin and Alexa Fluor 488 DNaseI (1:500; Molecular Probes) for 30 min at room temperature. For nuclear staining, cells were treated with 4′,6-diamidino-2-phenylindole. Coverslips were mounted onto microscope slides covered with antifade reagent. Images were captured using a Zeiss LSM510 META (Carl Zeiss Microscopy, Jena, Germany) confocal microscope.

Subcellular Fractionation Localization

The nuclear and cytosolic fractions were separated using the PARIS kit (Life Technologies, Thermo Fisher Scientific Inc., Waltham, MA, USA) according to the manufacturer's instructions. Then, RNA was extracted using TRIZOL (Invitrogen, Grand Island, NY, USA), and 2 μg of total RNA was reverse-transcribed using random primers and SuperScript III Reverse Transcriptase (Invitrogen, Grand Island, NY, USA). Finally, RT-PCR was performed to determine the expression level of LOC441461. U6 was used as the nuclear marker, and GAPDH was used as the cytosolic fraction marker.

U6-F: 5′-CTCGCTTCGGCAGCACA-3′;
U6-R: 5′-AACGCTTCACGAATTTGCGT-3;
GAPDH-F: 5′-TGCACCACCAACTGCTTAGC-3′;
GAPDH-R: 5′-GGCATGGACTGTGGTCATGAG-3′;
LOC441461-F: 5′-TGATAAGCTGTTTAAACCAGAACCG-3′; and
LOC441461-R: 5′-GGCAACATTTCAGTTCCAGTG-3′.

Wound Healing Assay

For the wound healing assay, after transfection with si-LOC441461 or negative control for 72 h, cells (1.5 × 10^6) were seeded on six-well plates. A straight line was scratched on the monolayer in the middle of the well by using the tip of a 200 mL pipette. Culture medium comprising 10% FBS was replaced with serum deprivation culture medium, and cells were then incubated at 37 °C. Wound closure was monitored and photographed at different time points under a microscope. Subsequently, the open area was assessed for quantifying cell migration ability.

© 2020 by the authors. Licensee MDPI, Basel, Switzerland. This article is an open access article distributed under the terms and conditions of the Creative Commons Attribution (CC BY) license (http://creativecommons.org/licenses/by/4.0/).
